# Convergent evolution of semiochemicals across Kingdoms: bark beetles and their fungal symbionts

**DOI:** 10.1038/s41396-019-0370-7

**Published:** 2019-02-15

**Authors:** Tao Zhao, Suresh Ganji, Christian Schiebe, Björn Bohman, Philip Weinstein, Paal Krokene, Anna-Karin Borg-Karlson, C. Rikard Unelius

**Affiliations:** 1 Department of Chemistry and Biomedical Sciences, Faculty of Health and Life Sciences, Linnaeus University, 382 91, Kalmar, Sweden; 2 Department of Chemistry, School of Engineering Sciences in Chemistry, Biotechnology and Health, Royal Institute of Technology, 100 44, Stockholm, Sweden; 3 School of Science and Technology, Örebro University, 701 82, Örebro, Sweden; 4 School of Molecular Sciences, University of Western Australia, Perth, Australia; 5 School of Biological Sciences, University of Adelaide, Adelaide, Australia; 6 Department of Plant Molecular Biology, Norwegian Institute of Bioeconomy Research, 1431 Ås, Norway

**Keywords:** Forest ecology, Fungal ecology, Metabolomics

## Abstract

Convergent evolution of semiochemical use in organisms from different Kingdoms is a rarely described phenomenon. Tree-killing bark beetles vector numerous symbiotic blue-stain fungi that help the beetles colonize healthy trees. Here we show for the first time that some of these fungi are able to biosynthesize bicyclic ketals that are pheromones and other semiochemicals of bark beetles. Volatile emissions of five common bark beetle symbionts were investigated by gas chromatography-mass spectrometry. When grown on fresh Norway spruce bark the fungi emitted three well-known bark beetle aggregation pheromones and semiochemicals (*exo*-brevicomin, *endo*-brevicomin and *trans*-conophthorin) and two structurally related semiochemical candidates (*exo*-1,3-dimethyl-2,9-dioxabicyclo[3.3.1]nonane and *endo*-1,3-dimethyl-2,9-dioxabicyclo[3.3.1]nonane) that elicited electroantennogram responses in the spruce bark beetle *Ips typographus*. When grown on malt agar with ^13^C d-Glucose, the fungus *Grosmannia europhioides* incorporated ^13^C into *exo*-brevicomin and *trans*-conophthorin. The enantiomeric compositions of the fungus-produced ketals closely matched those previously reported from bark beetles. The production of structurally complex bark beetle pheromones by symbiotic fungi indicates cross-kingdom convergent evolution of signal use in this system. This signaling is susceptible to disruption, providing potential new targets for pest control in conifer forests and plantations.

## Introduction

Symbiotic interactions between insects and microorganisms are widespread in nature. There is growing evidence that microbial symbionts play instrumental roles in animal ecology [1–3] and many of these roles are potentially mediated by microbe-produced chemical signals [4]. It has been noted that many insects with aggregation behavior are closely associated with specific microbial communities [5–10]. A potential explanation for these specific insect-microbe associations can involve the convergent synthesis of chemical communication signals (semiochemicals) across Kingdoms [11, 12].

A small subset of the world’s 6000 bark beetle species are aggressive tree-killers, and these tree-killing species are some of the most devastating pests in conifer forests worldwide. The key to the beetles’ ability to kill trees seems to be their complex chemical communication system and symbiotic relationship with phytopathogenic blue-stain fungi [13, 14]. Bark beetles use chemical cues to distinguish suitable hosts [15]. As soon as pioneer beetles land on a suitable host, they release aggregation pheromones that may coordinate a deadly mass attack on the tree [13, 16]. When the number of invaders reaches a critical level, the beetles release anti-aggregation pheromones to repel newcomers and limit intraspecific competition in the bark [13, 16]. Fungi carried externally and in the beetle gut increase the virulence of each beetle attack and help neutralize the trees’ defenses [17, 18]. Thus, the association between bark beetles and their symbiotic fungi seems to increase the fitness of both partners. The symbiotic relationship is obligate in so far as the fungi depend on the beetle for their own dispersal [19]. It would be adaptive, therefore, for the fungi and the beetle to use mutually intelligible signaling systems, despite belonging to different biological Kingdoms.

The phytopathogenic blue-stain fungi associated with tree-killing bark beetles belong to *Endoconidiophora*, *Ophiostoma*, *Grosmannia*, and related genera [20, 21]. Blue-stain fungi are chemically versatile organisms that effectively metabolize phenolic and terpenoid compounds in conifers [18, 22, 23]. Recently, these fungi have been found to produce volatile compounds that act as semiochemicals for several bark beetle species. These compounds have all been structurally relatively simple alcohols, acetates and terpenoids [24]. For example, in an exploratory study we found that the fungal symbionts of the spruce bark beetle (*Ips typographus*) produce the tertiary alcohol 2-methyl-3-buten-2-ol, a key component of the beetle’s aggregation pheromone blend [25].

In contrast to the structurally simple pheromone compound 2-methyl-3-buten-2-ol, we report here that some beetle-associated fungi produce multiple bicyclic ketals of fatty-acid origin that play essential roles in the chemical communication of many bark beetle species [26–28]. Among these structurally complex compounds are *exo*-brevicomin and *endo*-brevicomin (7-ethyl-5-methyl-6,8-dioxabicyclo[3.2.1]octane) that are important attractants produced by males of the European species *Dryocoetes autographus* [27]. (+)-*exo*-Brevicomin is an aggregation pheromone component released by female western pine beetles (*Dendroctonus brevicomis*) [29] and male mountain pine beetles (*Dendroctonus ponderosae*) [30], two important tree-killers in North America. The (+)-enantiomer of *endo*-brevicomin is a key component in the aggregation pheromone of the southern pine beetle (*Dendroctonus frontalis*) [31]. Another bicyclic ketal, (5*S*,7*S*)-*trans*-conophthorin ((*E*)-7-methyl-1,6-dioxaspiro-[4.5]decane), is best known as a non-host volatile serving as an anti-attractant in several economically important bark beetle species, including *I. typographus* [28, 32, 33].

The biosynthesis of bicyclic ketals is relatively well studied in bark beetles but much less is known about fungi. In *D. ponderosae*, *exo*-brevicomin has been shown to be derived de novo from oxidation of mono-unsaturated fatty acids and subsequent P450-mediated conversion of (*Z*)-6-nonen-2-one in the beetle fat body [34, 35]. In fungi, the biosynthesis of bicyclic ketals is largely unknown [36], although several fungi have been shown to produce conophthorin when linoleic acid is used as the growth medium [37]. The limited information available suggests that bark beetles and fungi use different biosynthethic pathways to produce bicyclic ketals and that fungi use a more complicated pathway involving oxidation of poly-unsaturated fatty acids which requires additional enzymes [37].

**Fig. 1:**
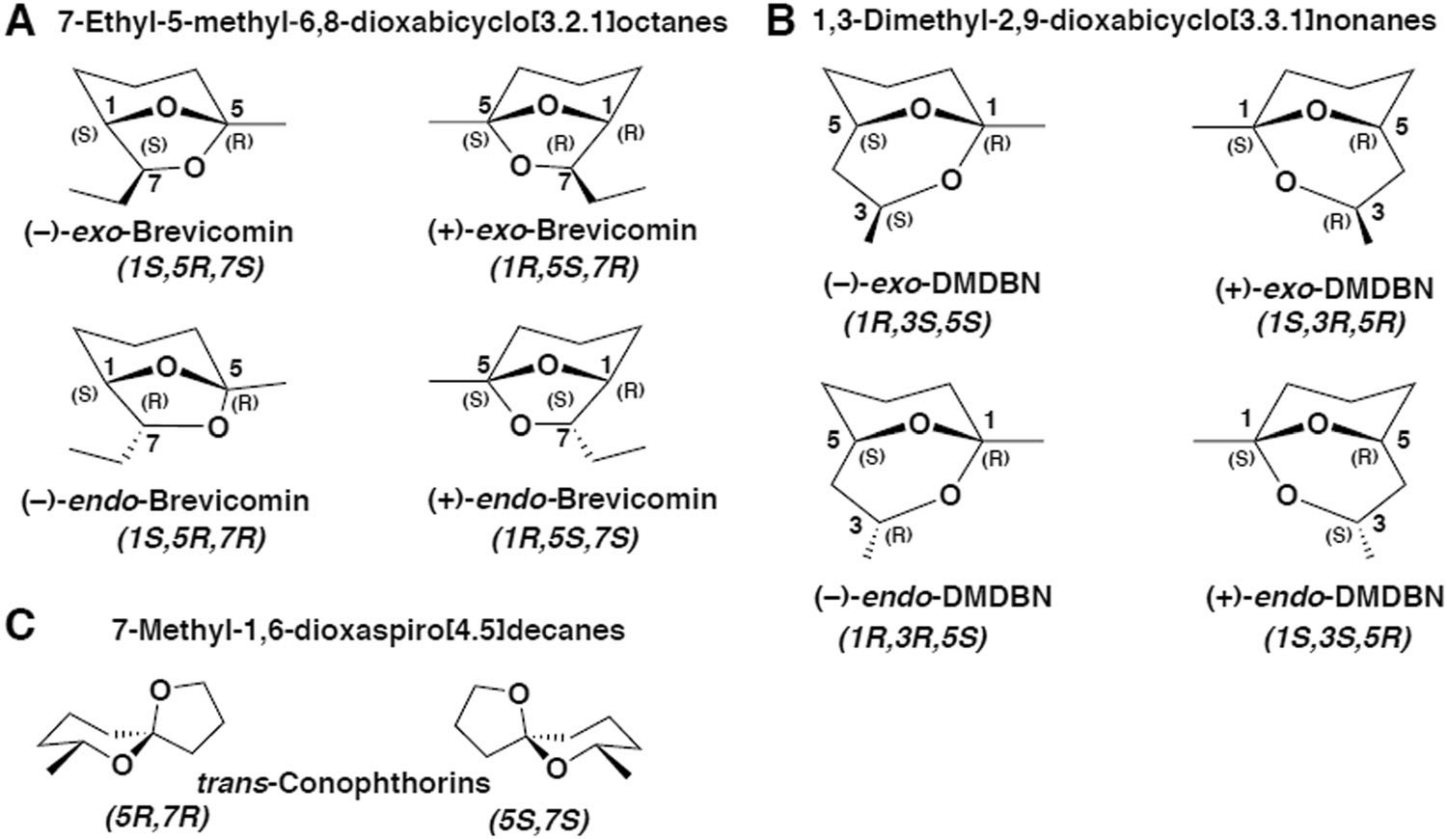
Structures of bicyclic ketals detected from cultures of bark beetle-associated blue-stain fungi: **a** brevicomin, **b** 1,3-dimethyl-2,9-dioxa-bicyclo[3.1.1]nonane (1,3-DMDBN), **c** *trans*-conophthorin

In this study, we investigated the production of bicyclic ketals by five common fungal symbionts of European bark beetles: *Endoconidiophora polonica, Grosmannia europhioides*, *G. penicillata, Ophiostoma bicolor*, and *O. piceae*. The products detected included three well-known bark beetle semiochemicals (*exo*-brevicomin, *endo-*brevicomin and (5*S*,7*S*)-*trans*-conophthorin) and two structurally related compounds (*exo*-1,3-dimethyl-2,9-dioxabicyclo[3.3.1]nonane (1,3-DMDBN) and *endo*-1,3-DMDBN) (Fig. 1) that elicited antennal responses in *I. typographus*. The production of structurally complex bark beetle pheromones and other possible semiochemicals by symbiotic fungi indicates cross-kingdom convergent evolution of signal use in bark beetles and blue-stain fungi.

## Materials and methods

### Fungal symbionts

Five common fungal symbionts of European bark beetles, *Endoconidiophora polonica, Grosmannia europhioides*, *G. penicillata, Ophiostoma bicolor*, and *O. piceae*, were used in this study. *Endoconidiophora polonica* is mainly associated with the spruce bark beetle, while the other four fungal species are associated with multiple bark beetle species (Table S1) [38–43]. All fungal isolates were obtained from the culture collection of the Norwegian Institute of Bioeconomy Research in Ås, Norway. Isolates were maintained on malt agar (2% malt, 1.5% agar) at 4 °C, and transferred to fresh malt agar and cultivated at 25 °C in darkness for 7–10 days before the start of the experiments. For more details on the isolates used and the biology of the species, see Table S1 and Zhao et al. [18, 25].

### Bioassays to detect bicyclic ketal production by fungi in spruce bark

We assayed the fungi’s ability to produce bicyclic ketals and other volatiles using the method described by Zhao et al. [25]. Bark plugs (10 mm diameter) with a pre-drilled hole (4 mm) in the center were taken from a fresh spruce log and placed individually in screw-top glass headspace vials (20 ml, Supelco, PA, USA). A plug (4 mm diameter) of sterile malt agar or malt agar colonized by one of the five fungi was inserted into the center of the bark plug to allow the fungi to colonize the bark. A total of 36 vials were prepared, with six replicates for each of the six treatments (agar with actively growing fungal mycelium of the five fungi and sterile agar control). When the bark plugs with agar or fungus had been loaded into the vials, the vials were immediately sealed by a stainless-steel cap with a polytetrafluoro-ethylene (PTFE)-faced butyl septum (Supelco, PA, USA) and incubated at 25 °C in darkness. Volatiles present in the headspace of each vial were collected for gas chromatography-mass spectrometry (GC-MS) analysis every 2 days for 9 days. Two days after GC-MS analysis was completed, the bark plugs were extracted individually in 0.5 ml hexane for 24 h for GC-electroantennogram detection (GC-EAD) analysis.

### Bioassays on malt agar with ^13^C labeled glucose

To confirm that any bicyclic ketals detected in the bark plug incubation assay were produced de novo by fungi, we cultivated *G. europhioides* (a fungus that produced all bicyclic ketals on spruce bark) on malt agar (2% malt, 1.5% agar) spiked with 0.5% d-Glucose (>99.5%, Sigma-Aldrich, MO, USA) or 0.5% ^13^C labeled d-Glucose (99% U-^13^C_6_, Cambridge Isotope Laboratories, MA, USA) in 20 ml headspace vials. After fungal inoculation, all vials were sealed as above and kept at 25 °C for one week. Volatiles present in the headspace of each vial were then collected and analyzed by GC-MS (see below). The incorporation of ^13^C into bark beetle semiochemicals by *G. europhioides* was confirmed by comparing mass spectra of compounds collected from fungi growing on malt agar medium with and without ^13^C labeled glucose.

### Volatile collection and GC-MS analysis

Volatiles present in the headspace of each vial were collected for 50 min using a Gerstel multipurpose sampler equipped with a solid phase micro-extraction (SPME) device with a 65 µm polydimethylsiloxane/divinylbenzene (PDMS/DVB) fiber (Supelco, PA, USA). Immediately after SPME collection, volatiles were analyzed using the method described by Zhao et al. [25]. Briefly, the SPME fiber was inserted into the split/splitless injector of the GC-MS with a 30 s splitless injection at 225 °C for 5 min. Volatiles were analyzed using an Agilent 7890 GC combined with a 5975C MS with a triple-axis detector and an HP-5 capillary column (30 m × 0.25 mm inner diameter, 0.25 μm film thickness) (Agilent Technologies, CA, USA). Helium was used as the carrier gas at a constant flow of 1 ml/min. The temperature of the ion source was 150 °C and the electron impact ionization was 70 eV. The mass detector was operated with a mass range of 30–400 *m/z*. The oven program was 40 °C for 3 min, increasing by 4 °C min^−1^ to 160 °C, then by 20 °C min^−1^ to 230 °C and held constant for 5 min. Bark beetle semiochemicals were identified by comparison with published mass spectra [26] and confirmed by comparing retention times and mass spectra with synthesized reference compounds. The amounts of the detected compounds were calculated from the peak areas of the total ion chromatograms (TIC).

To determine the enantiomeric composition of the chiral compounds, we collected volatiles released by *G. europhioides* growing on malt agar and in spruce bark. Volatiles were collected at five time-points 2–15 days after fungal inoculation (three replicates per time point) to monitor the enantiomeric composition over time. Enantiomers were detected using an Agilent 6890 GC and a 5973 MSD (Hewlett Packard, CA, USA) with an enantioselective capillary column (CyclosilB, 30 m × 0.25 mm inner diameter, 0.25 μm film thickness) (J&W Scientific, CA, USA). Helium was used as a carrier gas at a constant flow of 1 ml min^−1^. The mass detector was operated with a mass range of 30–400 *m/z* at 70 eV. The temperature program started at 40 °C for 3 min, increasing by 3 °C min^−1^ to 150 °C, then by 15 °C min^−1^ to 250 °C and held constant for 10 min. The enantiomers of the bicyclic ketals released by the fungi were verified by co-elution with enantiomerically enriched synthetic reference compounds on a GC-column with the chiral stationary phase described above.

### Synthesis and enantiomeric purification of bicyclic ketals

All isomers of brevicomins (Fig. 1a), were synthesized from methyl (*R*)-2-hydroxybutanoate and methyl (*S*)-2-hydroxybutanoate via intermediate non-8-ene-2,4-diols (Fig. S1) [44, 45]. By analogy, 1,3-dimethyl-2,9-dioxabicyclo[3.3.1]nonanes (1,3-DMDBNs) (Fig. 1b) were synthesized from methyl (*R*)-3-hydroxybutanoate and methyl (*S*)-3-hydroxybutanoate via non-8-ene-3,4-diols (Fig. S1) [45, 46]. A racemic mixture of conophthorin diastereomers (*trans*:*cis* = 95:5) was provided by Syntastic AB, Sweden and the enantiomers (Fig. 1c) were separated on an enantioselective GC column as described above. The elution order was determined with the help of a reference of (5*S*,7*S*)-*trans*-conophthorin obtained from Prof W. Francke (University of Hamburg) via SLU, Alnarp, Sweden.

### Combined gas chromatography and electroantennogram detection (GC-EAD)

We used GC-EAD to determine antennal responses of the spruce bark beetle to *exo-*1,3-DMDBN and *endo*-1,3-DMDBN, following the method described by Schiebe [47]. In brief, an IDAC-2 (Syntech, Kirchzarten, Germany) was coupled to an Agilent 6890 GC with a HP-5 column (Agilent Technologies, CA, USA) and a FID detector. Hexane extracts (2 µl) of the fungus-infested bark plugs from the headspace vials were injected manually into the GC injector in splitless mode (0.5 min). The injector temperature was 225 °C, and the oven temperature program was 50 °C for 3 min, increasing by 5 °C min^−1^ to 150 °C, held for 3 min at 150 °C, followed by an increase of 8 °C min^−1^ to 250 °C, and a final increase of 15 °C min^−1^ to 325 °C. Spruce bark beetle antennae were prepared according to Zhang et al. [48] and mounted as close as possible to the outlet of the glass tube. Replicates of four beetles were used to verify each response. Recordings were obtained and assessed using the software Syntech GC-EAD versions 1.1 and 1.2.3.

### Data analysis

Amounts of ketals released by different fungi were subjected to a  repeated measures one-way ANOVA (Statistica 6.0, Statsoft Inc., USA). Data were log (*X* + 1) transformed to correct for unequal variance and departures from normality, and treatment means were separated using Tukey HSD post hoc test at *p* = 0.05.

## Results

### Bicyclic ketal production by blue-stain fungi growing on spruce bark

**Fig. 2:**
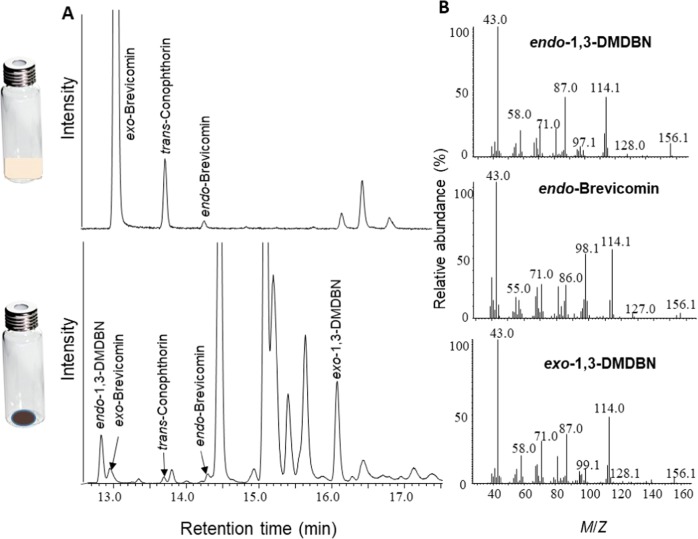
**a** Representative chromatograms showing bicyclic ketals released from the blue-stain fungus *Grosmannia europhioides* growing on malt agar (top) or fresh spruce bark (bottom). *Exo*-brevicomin, *endo*-brevicomin and *trans*-conophthorin were detected from both growth media, whereas *exo*-1,3-dimethyl-2,9-dioxabicyclo[3.1.1]nonane (1,3-DMDBN) and *endo*-1,3-DMDBN were detected exclusively from the bark. **b** Mass spectra of *endo*-brevicomin, *exo*-1,3-DMDBN and *endo*-1,3-DMDBN. For mass spectra of *exo*-brevicomin and *trans*-conophthorin, see Fig. 3

**Fig. 3:**
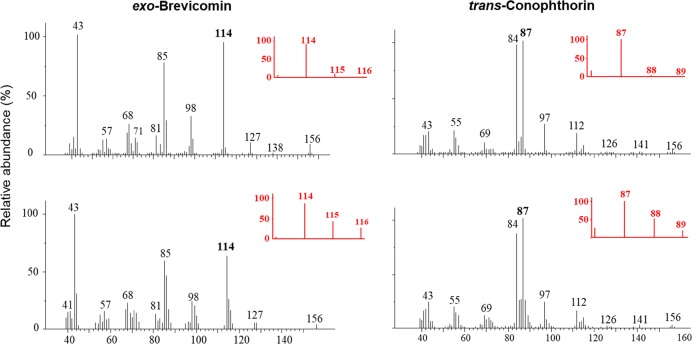
Representative mass spectra showing incorporation of ^13^C into *exo*-brevicomin and *trans*-conophthorin produced by the blue-stain fungus *Grosmannia europhioides*. Upper and lower mass spectra represent fungus growing on malt agar with 0.5% unlabeled d-Glucose or ^13^C labeled d-Glucose (U-^13^C6), respectively. Red enlargements of spectra show representative fragments labeled by ^13^C

**Fig. 4:**
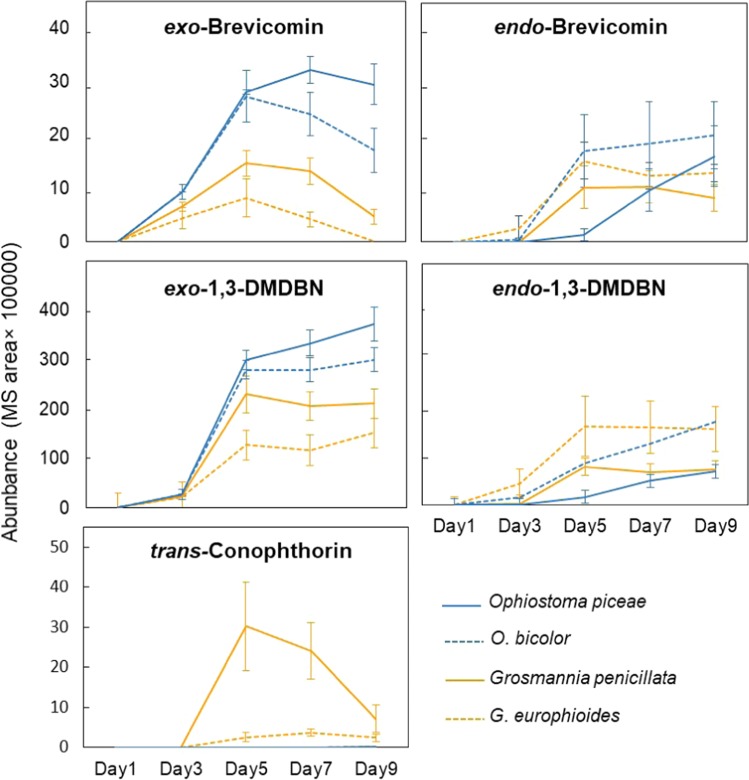
Abundance of five bicyclic ketals released from Norway spruce bark incubated with sterile malt agar (control) or different blue-stain fungi associated with European bark beetles. Data are expressed as mean abundance ± 1 SD 1–9 days after incubation (*N* = 6). 1,3-DMDBN represents 1,3-dimethyl-2,9-dioxabicyclo[3.3.1]nonanes. No ketals were detected from control bark or bark incubated with *Endoconidiophora polonica*

To determine if blue-stain fungi could produce bicyclic ketals while growing on fresh Norway spruce bark we analyzed volatile emissions from five common fungi associated with bark beetles: *Endoconidiophora polonica, Grosmannia europhioides*, *G. penicillata, Ophiostoma bicolor*, and *O. piceae*. Un-colonized control bark plugs and bark plugs colonized by *E. polonica* emitted mostly mono- and sesquiterpenes originating from the bark. However, bark plugs colonized by the four other fungi emitted a more complex mixture of volatile compounds, including five bicyclic ketals (Fig. 2 and 3). All fungi except *E. polonica* emitted *exo*-brevicomin, *endo*-brevicomin, *exo*-1,3-DMDBN and *endo*-1,3-DMDBN. The two *Grosmannia* species also emitted (*5S,7S*)-*trans*-conophthorin (Fig. 4).

The amount of emitted *endo*-brevicomin did not differ significantly among *G. europhioides, G. penicillata, O. bicolor* and *O. piceae* at any sampling time (*p* > 0.09), but the two *Ophiostoma* species emitted significantly more *exo*-brevicomin than the two *Grosmannia* species (*p* < 0.01). For example, 7 days after inoculation, *O. piceae* emitted seven times more *exo*-brevicomin than *G. europhioides*, and three times more than *G. penicillata* (Fig. 4). Consequently, the two *Ophiostoma* species emitted a significantly higher ratio of *exo*-brevicomin to *endo*-brevicomin than did the *Grosmannia* species (*p* < 0.03).

All *Grosmannia* and *Ophiostoma* species released about 10 times more 1,3-DMDBN than brevicomin (Fig. 4). For 1,3-DMDBN, the four fungi released similar amounts of the *endo*-isomer at all sampling times (*p* > 0.11), but the *Ophiostoma* species released more *exo*-1,3-DMDBN than the *Grosmannia* species (*p* < 0.05) (Fig. 4).

Unlike the other ketals, (*5S,7S*)-*trans*-conophthorin was only released by the two *Grosmannia* species: *G. penicillata* released seven times more *trans*-conophthorin than *G. europhioides* in total over the 9-day sampling period, with a peak on day 5 when it released 12 times more *trans-*conophthorin (Fig. 4).

### De novo bicyclic ketal production by *Grosmanniaeurophioides*

To determine if bark beetle-associated blue-stain fungi could produce bicyclic ketals de novo, we investigated volatiles present in the headspace of malt agar cultures of *G. europhioides*, a species that produced all five detected ketals when growing on spruce bark. When growing on malt agar, the fungus produced *exo*-brevicomin and *trans*-conophthorin, but no 1,3-DMDBNs and only minute amounts of *endo*-brevicomin (Fig. 2). When *G. europhioides* was incubated on malt agar containing 0.5% ^13^C labeled d-Glucose, a clearly visible incorporation of ^13^C into *exo*-brevicomin and (*5S,7S*)-*trans*-conophthorin was detected in the headspace of the fungus 7 days after incubation (Fig. 3). This is conclusive evidence that *G. europhioides* can produce these compounds de novo.

### Enantiomeric composition of fungus-produced bicyclic ketals

The enantiomeric composition of the bicyclic ketals released by *G*. *europhioides *were determined by co-injection of synthetic standards with fungal samples in enantioselective gas chromatography (GC) (Fig. S2). *Grosmannia europhioides* released mainly (+)-*exo*-brevicomin (>94%) and (5*S*,7*S*)-*trans*-conophthorin (>94%), with minor amounts of (−)-*exo*-brevicomin (<6%) and (5*R*,7*S*)-*cis*-conophthorin (<6%). When growing on spruce bark (Fig. S2), *G. europhioides* also released enantiomerically pure (>99%) (−)-*exo*-1,3-DMDBN and (>96%) (−)-*endo*-1,3-DMDBN constantly over the whole sampling period.

### Antennal responses in the spruce bark beetle

**Fig. 5:**
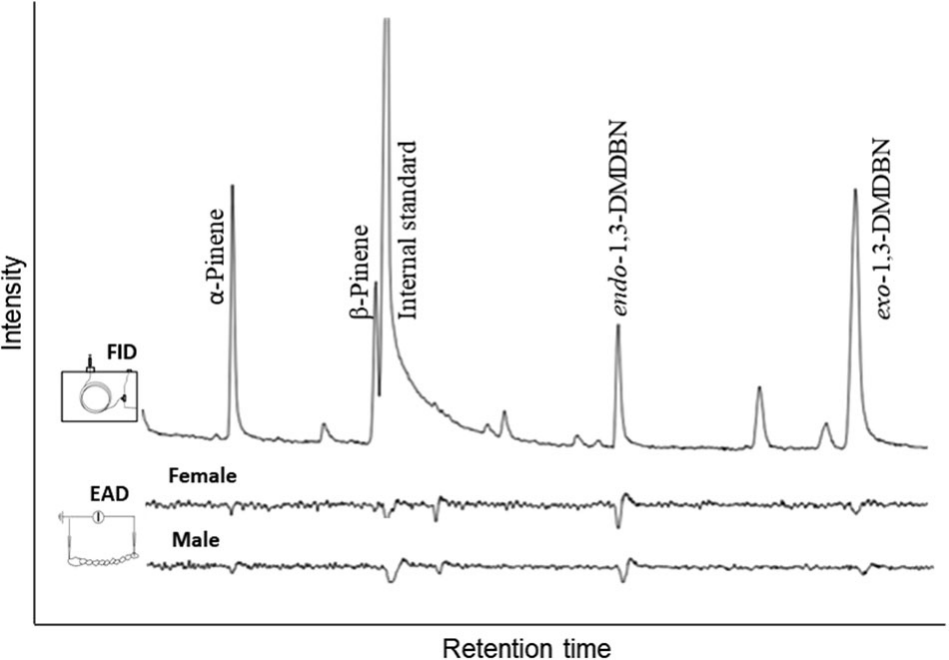
Representative GC-EAD recordings showing that both male and female *Ips typographus* have a clear antennal response towards *exo*-1,3-dimethyl-2,9-dioxa-bicyclo[3.1.1]nonane (1,3-DMDBN) and *endo*-1,3-DMDBN presented in hexane extracts of bark plugs inoculated with the blue-stain fungus *Grosmannia europhioides*. Extractions were conducted 11 days after fungal inoculation

To determine whether the spruce bark beetle could percieve *exo*-1,3-DMDBN and *endo*-1,3-DMDBN we measured the antennal response by *I. typographus* to these compounds using combined gas chromatography and electroantennogram detection (GC-EAD). Highly repeatable EAD responses (using an achiral GC-column) were recorded to both compounds present in hexane extracts of *G. europhioides-*infested bark. The compounds induced antennal responses in both male and female beetles, with the *endo*-isomer eliciting the strongest responses (Fig. 5).

## Discussion

In this study we show an example of cross-kingdom convergent evolution of semiochemical use involving symbiotic fungi producing aggregation pheromones, anti-aggregation pheromones, and spacing semiochemicals of several primary and secondary bark beetle species. The convergent evolution of semiochemical use between organisms of different kingdoms is a rarely described phenomenon. Best known are perhaps the sexually deceptive orchids that hijack the pollination services of male wasps by emitting identical semiochemicals to those of the female wasps [11, 12]. However, there are other intriguing examples of convergent evolution that have evolved under completely different ecological selection pressures, including defense against herbivory. Plant cyanogenic glucosides are highly toxic anti-feedants that are sometimes sequestered by caterpillars for their own anti-predator defense [49]. Such anti-predator defense provides a sufficiently strong selective advantage that the Burnet moth (*Zyaena filipenduale*) has evolved the ability not only to sequester linamarin and lotaustralin from its host plant *Lotus corniculatus*, but also to synthesize these compounds de novo [50].

In our study system, the association between bark beetles and blue-stain fungi benefits both parties. Blue-stain fungi depend on bark beetles for their dispersal, whereas fungi increase the efficacy of beetle attacks since the fungi help to neutralize tree defenses [17, 18]. This mutual benefit suggests that it would be a selective advantage for fungi and beetles to use mutually intelligible signaling systems. The production of the same chemical signals by blue-stain fungi and insects in our case, is most probably not a synplesiomorphy (an ancestral trait shared by insects and fungi), but rather a homoplasy (convergent evolution of similar traits between species with no recent common ancestor). If biosynthesis of bicyclic ketals was an ancestral trait present in the common ancestor of fungi and animals we would expect to find this trait in many or most extant species of fungi and animals, but this is not the case. The available information suggests that bicyclic ketal production occurs sporadically among fungi, insects, plants and mammals [28, 29, 37, 51–53]. The fact that fungi and insects appear to use different biosynthetic pathways to produce bicyclic ketals [34, 37] also suggest that de novo synthesis of bicyclic ketals in blue-stain fungi and bark beetles is an example of convergent evolution and not a synplesiomorphy. The bark beetle *D. ponderosae* utilizes mono-unsaturated palmitic and oleic acid to produce *exo*-brevicomin [34]. Spores of five different fungal species produced no bicyclic ketals when provided with these fatty acids, but produced spiroketals on poly-unsaturated linoleic and linolenic acid media [37]. We therefore infer that we have established yet another example of the rarely documented phenomenon of convergent evolution of semiochemicals across kingdoms.

There is increasing evidence that volatile metabolites of microbial symbionts act as signals to insects, providing information about various aspects of habitat suitability [3, 4]. In bark beetle-fungal systems, it is known that volatiles released by *Ophiostoma novo-ulmi* (the pathogen causing Dutch elm disease) are attractive to its American vector, the elm bark beetle (*Hylurgopinus rufipes*) [54]. The redbay ambrosia beetle (*Xyleborus glabratus*) and three co-occurring beetle species (*Xyleborus ferrugineus*, *Xylosandrus crassiusculus* and *Xyleborinus saxesenii*) are attracted to the odors of their symbiotic fungal species [55]. Similarly, the parasitoid pteromalid wasp (*Heydenia unica*) attacking the larvae of the pine engraver beetle (*Ips pini*) can exploit volatiles released by the blue-stain fungus *Ophiostoma ips* to locate its bark beetle host [56]. However, the chemical basis underlying these different interactions is largely unknown. Our study provides the first evidence that blue-stain fungi can synthesize bicyclic ketals functioning as bark beetle aggregation pheromones, anti-aggregation pheromone or other semiochemicals.

Many *Dendroctonus*, *Dryocoetes*, and *Hylastes* bark beetle species synthesize *exo*-brevicomin and *endo*-brevicomin and use them as aggregation or anti-aggregation pheromones [51]. In Europe, males of *D. autographus* produce a blend of *exo*-brevicomin and *endo*-brevicomin that attracts both male and female beetles on Norway spruce [27]. Interestingly, there are also reports that *I. typographus* has olfactory receptor cells specific to *exo-*brevicomin [57, 58] and that this compound enhances the attraction of male beetles to the commercial pheromone blend Ipslure (consisting of ipsdienol, *cis*-verbenol and 2-methyl-3-butene-2-ol) [58]. *exo*-Brevicomin has also been found to be attractive to *I. typographus*, the six-spined spruce bark beetle(*Pityogenes chalcographus*) and other secondary bark beetle species in field experiments [59]. Collectively, these observations suggest that *exo*-brevicomin and *endo*-brevicomin produced by fungi may be used as interspecific chemical cues for host location by bark beetles in Norway spruce ecosystems.

*trans*-Conophthorin functions as a spacing signal that reduces attack density and competition in several conifer-infesting bark beetles in Europe, including *I. typographus*, the two-toothed pine beetle (*Pityogenes bidentatus*) and the twig beetle (*Pityophthorus pubescens*) [28, 60, 61]. This compound has been reported as a non-host volatile from broadleaf tree species [28], but has also been detected in male ash bark beetles (*Leperisinus varius*), in the frass of the fir bark beetle (*Cryphalus piceae*) [62] and from spores of almond-infecting and pistachio-infecting *Aspergillus*, *Penicillium* and *Rhizopus* fungi [37]. In our study, *trans*-conophthorin was only produced by the two *Grosmannia* species which are virulent tree pathogens that very effectively metabolize tree defense chemicals [18]. Seven times more *trans*-conophthorin was produced by *G. penicillata* than by its less virulent relative *G. europhioides*. A possible interpretation of this result is that virulent fungi have evolved volatile signaling mechanisms to reduce beetle density and thus diminish competition from other beetle-vectored fungal communities.

In addition to brevicomins and *trans*-conophthorin, we detected the bicyclic ketals *exo*-1,3-DMDBN and *endo*-1,3-DMDBN from spruce bark colonized by species of *Grosmannia* and *Ophiostoma*. Interestingly, both 1,3-DMDBN diastereomers induced antennal responses in both female and male *I. typographus*. The *endo-*isomer of 1,3-DMDBN has been suggested to be a host-specific substance, whereas the *exo*-isomer has not previously been identified from bark beetle systems [26]. We showed that spruce bark colonized by species of *Grosmannia* and *Ophiostoma* emitted both compounds, but the compounds were not detected from fungi growing on malt agar, control spruce bark, or bark colonized by *E. polonica*. This suggests that the tested *Grosmannia* and *Ophiostoma* species can produce these compounds from precursors present in spruce bark. Further studies are needed to explore whether these compounds induce behavioral responses in bark beetles.

Our ^13^C labeling studies unequivocally demonstrated that *G. europhioides* can produce (+)-*exo*-brevicomin and (*5S,7S*)-*trans*-conophthorin de novo. It is interesting to note from the experiments that the *exo-*diastereomer and *endo*-diastereomer of brevicomin might be produced by different biosynthetic pathways, as *G. europhioides* mainly produced *exo*-brevicomin when growing on malt agar, but required bark precursors to produce significant amounts of *endo*-brevicomin.

From our enantioselective GC analysis, it was evident that the enantioselectivity of the bicyclic ketals synthesized by *G. europhioides* closely matched the enantioselectivity of bark beetle- and plant-produced semiochemicals. The fungi produced (+)-*exo*-brevicomin and (*5S,7S*)-*trans*-conophthorin of high enantiomeric purity. This is in agreement with previous observations from the Western balsam bark beetle (*Dryocoetes confusus)* and the mountain pine beetle for brevicomin [63], and from angiosperm trees for *trans*-conophthorin [33]. The closely matched stereochemistry between plants, animals and fungi supports convergent evolution and possible signal appropriation between the organisms.

Blue-stain fungi are vectored by specialist or generalist bark beetles colonizing living trees or downed timber [20, 40, 64]. Sticky fungal spore masses carried on long-necked fruiting bodies provide a direct mechanism for the fungi to attach to the bodies of their vectors [10]. Blue-stain fungi have probably evolved the ability to produce volatile chemical signals to attract insect vectors that can transport the fungi to a new tree. In this study, *E. polonica* which is mainly associated with the spruce bark beetle [40, 41, 43], was the only fungus that did not produce any bicyclic ketals in either fresh spruce bark or malt agar. The ketal-producing *Ophiostoma* and *Grosmannia* species we studied are, on the other hand, associated with a range of bark beetle vectors (Table S1) [38–40, 42, 43] including *D*. *autographus* [42, 43, 65], a European bark beetle species that produces and uses *exo*-brevicomin and *endo*-brevicomin in its intraspecific communication [19, 49]. In central Europe, 44% of the examined *D. autographus* carry *O*. *piceae* [42], suggesting a close association between the beetle and the fungus. Interestingly, *O. piceae*, which is associated with a wide range of bark beetle species in Europe and North America [42, 43, 66], produced the highest amounts of *exo*-brevicomin in our study. These observations suggest that production of *exo*-brevicomin and *endo*-brevicomin by fungi may enhance their likelihood to be transported to host trees by multiple bark beetle species. However, the fact that the two studied *Grosmannia* species also produced *trans*-conophthorin, an anti-attractant and spacing signal for different bark beetles, suggests that interactions between *Grosmannia* blue-stain fungi and beetles may be more complex. More in-depth studies considering the dose and proportion of the compounds are needed to evaluate the behavioral effects of fungal volatile mixtures on bark beetles and other insects.

In conclusion, this study provides the first evidence that blue-stain fungi can biosynthesize bicyclic ketals acting as bark beetle pheromones and other semiochemicals. These results confirm the outcome of our exploratory study of blue-stain fungi producing the key pheromone component of the spruce bark beetle [25], and provide important new insights into beetle pheromone production as well as the interaction between bark beetles and symbiotic blue-stain fungi. Further, our findings support the hypothesis of convergent evolution of chemical signal use between blue-stain fungi and bark beetles. We demonstrate an intriguing commonality in chemical signals between species of different Kingdoms: by producing bark beetle semiochemicals, fungi probably increase the probability of their own survival and transmission to new hosts, while the bark beetles may use these compounds as chemical cues to find suitable hosts. The symbiotic relationship between beetles and fungi is intricate, and thus potentially vulnerable to disruption; our findings may, therefore, lay the foundation for new innovative pest management strategies using fungal volatiles.

## Supplementary information

Table S1

Figure S1

Figure S2
