# Supplementary material for: Convergent evolution of semiochemicals across Kingdoms: bark beetles and their fungal symbionts
Source: ISME J. 2019 Feb 15;13(6):1535–45. doi: 10.1038/s41396-019-0370-7 (PMC6776033; doi:10.1038/s41396-019-0370-7)
Supplement: Supplementary file 1 — Table S1 [file 41396_2019_370_MOESM1_ESM.docx]

Table S1: Fungal isolates used in this study. All isolates are from the culture collection of the Norwegian Institute of Bioeconomy Research

| Fungus | Isolate | Collected | Reported vectors | Plant hosts | References |
| --- | --- | --- | --- | --- | --- |
| *Endoconidiophora polonica*  (Siemaszko) de Beer, Duong & Wingf*.* | 1993-208/115 | 1993, Akershus (Ås)  Norway | *Ips typographus*  *Ips duplicatus* | *Picea abies* | [1, 2] |
| *Grosmannia europhioides*  (Wright & Cain) Zipfel, de Beer & Wingf. | 1990-119/20 | 1990, Nord-Trøndelag  Norway | *Dendroctonus valens*  *Dryocoetes autographus*  *Monochamus urussovi*  *Ips typographus*  *Pityogenes bidentatus*  *Polygraphus proximus* | [*Abies sibirica*](https://www.google.se/url?sa=t&rct=j&q=&esrc=s&source=web&cd=8&cad=rja&uact=8&ved=0ahUKEwirtajm35bYAhVDQpoKHYX4CJAQFgg6MAc&url=https%3A%2F%2Fen.wikipedia.org%2Fwiki%2FAbies_sibirica&usg=AOvVaw2D2UVV9ERpUx8ZHNUcbjBT)  *Picea abies*  *Picea obovata* | [1, 3, 4, 5] |
| *Grosmannia penicillata*  (Grosmann) Goid. | 2006-209/44/2 | 2006, Kronoberg (Växjö) Sweden | *Hylurgops palliates*  *Hylastes ater*  *Hylastes brunneus*  *Hylastes cunicularius*  *Ips amitinus*  *Ips duplicatus*  *Ips typographus*  *Pityogenes chalcographus*  *Pityogenes quadridens*  *Polygraphus poligraphus*  *Tomicus piniperda*  *Trypodendron lineatum* | *Picea abies*  *Pinus sylvestris*  *Picea obovata* | [1, 3, 6] |
| *Ophiostoma bicolor*  Davidson & Wells | 1980-48/36 | 1980, Akershus (Ås)  Norway | *Ips amitinus*  *Ips cembrae*  *Ips typographus*  *Ips duplicatus*  *Pityogenes chalcographus*  *Polygraphus poligraphus*  *Pityogenes quadridens* | *Picea abies*  *Pinus sylvestris*  *Larix sibirica*  *Picea obovata* | [1, 6, 7] |
| *Ophiostoma piceae*  (Műnch) Syd & Syd | 1986-432/3 | 1986, Akershus (Ås)  Norway | *Dryocoetes autographus Hylurgops palliates*  *Hylastes ater*  *Hylastes cunicularius*  *Ips amitinus*  *Ips typographus*  *Monochamus urussovi*  *Pityogenes chalcographus*  *Polygraphus poligraphus*  *Tomicus minor*  *Tomicus piniperda*  *Trypodendron lineatum* | *Picea abies*  *Pinus sylvestris*  *Abies sibirica* | [1, 3, 7, 8] |

1. Kirisits T. Fungal associates of European bark beetles with special emphasis on the ophiostomatoid fungi. In: Lieutier F, Day KR, Battisti A, Gregoire JC, Evans H (eds). *Bark and wood boring insects in living trees in Europe, a synthesis,* 1st edn. Kluwer Academic Publishers: Dordrecht, NL, 2004, pp 181-235.

2. Harrington T, Pashenova N, McNew D, Steimel J, Konstantinov MY. Species delimitation and host specialization of *Ceratocystis laricicola* and *C. polonica* to larch and spruce. *Plant Dis* 2002; **4**:418-422.

3. Linnakoski R, de Beer ZW, Duong TA, Niemelä P, Pappinen A, Wingfield MJ. *Grosmannia* and *Leptographium* spp. associated with conifer-infesting bark beetles in Finland and Russia, including *Leptographium taigense* sp. nov. *Antonie Van Leeuwenhoek* 2012; **102**:375-399.

4. Yamaoka Y. Taxonomy and pathogenicity of ophiostomatoid fungi associated with bark beetles infesting conifers in Japan, with special reference to those related to subalpine conifers. *Mycoscience* 2017; **4**:221-235.

5. Wright EF, Cain RF. New species of the genus Ceratocystis. *Can. J. Bot.* 1961; **39**:1215-1230.

6. Linnakoski R, De Beer ZW, Niemelä P, Wingfield MJ. Associations of conifer-infesting bark beetles and fungi in Fennoscandia. *Insects* 2012; **1**:200-227.

7. Linnakoski R, De Beer ZW, Ahtiainen J, Sidorov E, Niemelä P, Pappinen A*, et al.* *Ophiostoma* spp. associated with pine- and spruce-infesting bark beetles in Finland and Russia. *Persoonia* 2010; **25**:72-93.

8. Jankowiak R, Strzałka B, Bilański P, Kacprzyk M, Lukášová K, Linnakoski R*, et al.* Diversity of Ophiostomatales species associated with conifer-infesting beetles in the Western Carpathians. *Eur J Forest Res* 2017; **5-6**:939-956.
