# Supplementary material for: Convergent evolution of semiochemicals across Kingdoms: bark beetles and their fungal symbionts
Source: ISME J. 2019 Feb 15;13(6):1535–45. doi: 10.1038/s41396-019-0370-7 (PMC6776033; doi:10.1038/s41396-019-0370-7)
Supplement: Supplementary file 2 — Figure S1 [file 41396_2019_370_MOESM2_ESM.docx]

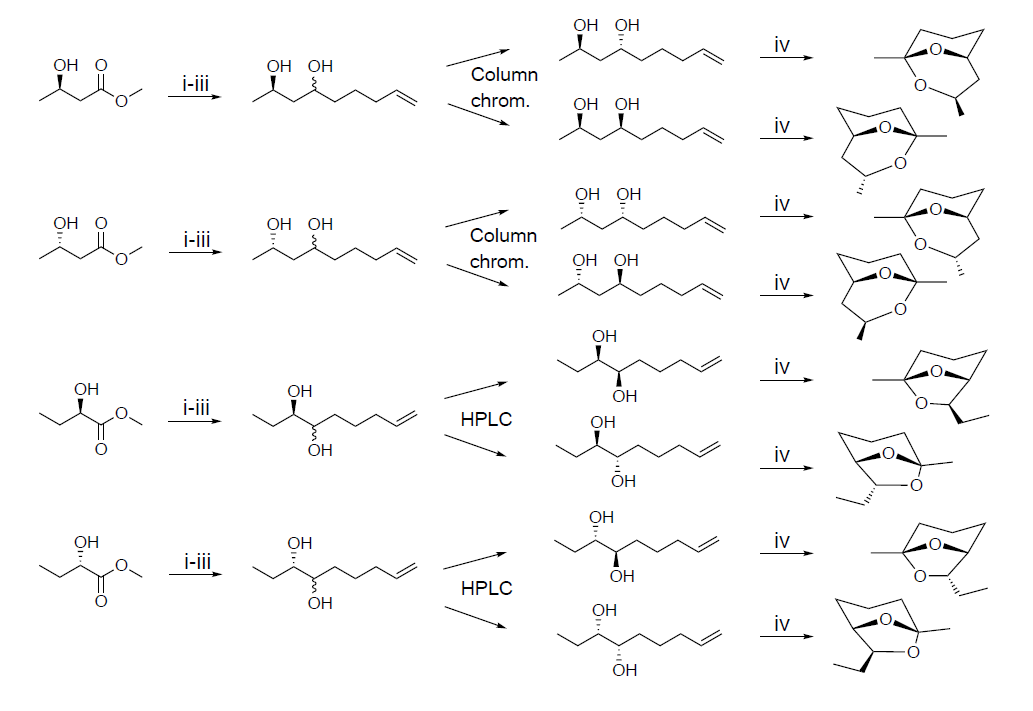


**Fig. S1** Scheme for the synthesis of brevicomins and 1,3-dimethyl-2,9-dioxa-bicyclo[3.1.1]nonanes. **i.** diisobutylaluminium hydride (DIBAL, 2 equiv. in toluene); **ii.** 4-pentenyl-magnesium bromide (2 equiv in THF/toluene); **iii.** H+/H2O; **iv.** PdCl2/CuCl2 x H2O in THF. All isomers of brevicomins were synthesized from methyl (*R*)-2-hydroxybutanoate and methyl (*S*)-2-hydroxybutanoate by DIBAL reductions followed by *in situ* Grignard reactions to yield the intermediate non-8-ene-2,4-diols, which diastereomers were chromatographically separated before Wacker oxidations and ketalisations to yield the enantiomerically pure brevicomins. 1,3-Dimethyl-2,9-dioxabicyclo[3.3.1]nonanes were synthesized from methyl (*R*)-3-hydroxybutanoate and methyl (*S*)-3-hydroxybutanoate via non-8-ene-3,4-diols.
