# Supplementary material for: Convergent evolution of semiochemicals across Kingdoms: bark beetles and their fungal symbionts
Source: ISME J. 2019 Feb 15;13(6):1535–45. doi: 10.1038/s41396-019-0370-7 (PMC6776033; doi:10.1038/s41396-019-0370-7)
Supplement: Supplementary file 3 — Figure S2 [file 41396_2019_370_MOESM3_ESM.docx]

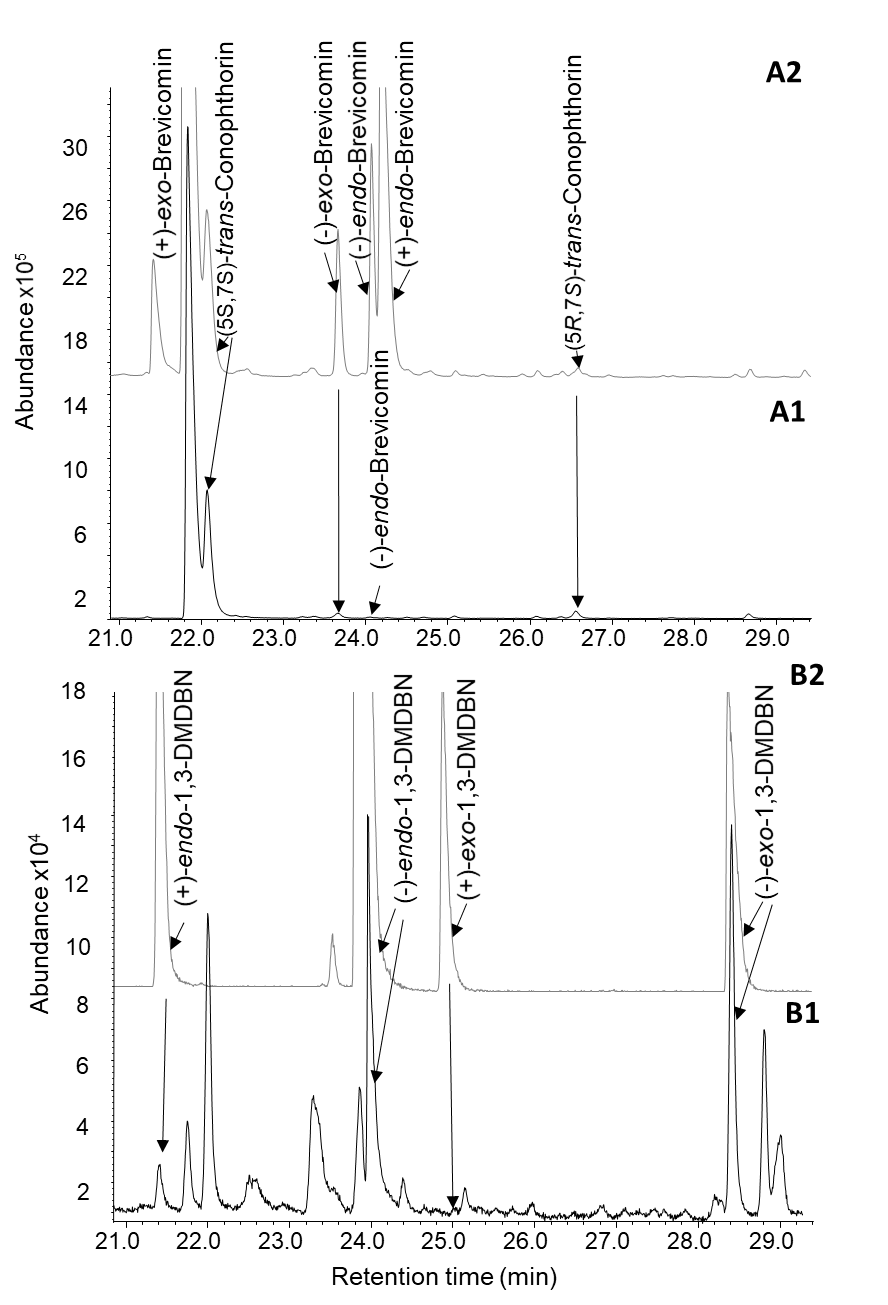


**Fig. S2** Representative chromatograms showing the separation of enantiomers of bicyclic ketals released by the blue-stain fungus *Grosmannia europhioides* growing on malt agar (A1) and Norway spruce bark (B1). A2 showing the co-elution of *G. europhioides* emission with conophthorin and brevicomin mixture on malt agar; B2 showing the co-elution of the fungus with 1,3-DMDBN (1,3-dimethyl-2,9-dioxa-bicyclo[3.1.1]nonane) on Norway spruce bark.
